# Computational Models in Non-Coding RNA and Human Disease

**DOI:** 10.3390/ijms21051557

**Published:** 2020-02-25

**Authors:** Xing Chen, Chun-Chun Wang, Na-Na Guan

**Affiliations:** 1 School of Information and Control Engineering, China University of Mining and Technology, Xuzhou 221116, China; ts17060063a3@cumt.edu.cn; 2 College of Big Data Statistics, Guizhou University of Finance and Economics, Guiyang 550025, China; gnn@mail.gufe.edu.cn

The central dogma of molecular biology has told that DNA sequences encode proteins through RNAs, which function as an information intermediary [1]. For decades, these protein-coding genes in the human genome have attracted considerable attentions from researchers. However, studies have shown that these genes merely account for about 1.5% to 3% of the whole human genome, while most of which consists of non-coding RNA (ncRNA) [2,3]. The first two ncRNAs were discovered in *Caenorhabditis elegans* in 1993 and 2000, respectively, which are *lineage defective 4* (*lin-4*) [4] and *lethal 7* (*let-7*) [5]. Since then, thousands of ncRNAs have been identified in various species due to the development of sequencing technology [6]. Based on the transcript length and structure, ncRNAs can be divided into different classes, including microRNA (miRNA), long non-coding RNA (lncRNA), transcribed ultraconserved regions (T-UCRs), small nucleolar RNAs (snoRNAs), PIWI-interacting RNAs (piRNAs) and circular RNA (circRNA) [7], etc.

Although the functions of many identified ncRNAs have not been well-studied, increasing experimental evidences have suggested that these ncRNAs are involved in multiple fundamental and important biological functions. It was shown that ncRNAs could regulate cellular processes and pathways associated with cell development and pathology [8]. For example, miRNAs regulate gene expression through binding of the seed sequence to the target messenger RNA (mRNA) [9], leading to translational repression or mRNA degradation [10], also increasing the mRNA translation and protein output in some cases [11,12,13]. Moreover, lncRNAs have been found to be implicated in almost every process of the cellular life cycle [14] and perform diverse critical biological functions [15]. LncRNAs can regulate transcriptional and posttranscriptional processes, participate in epigenetic regulation, and therefore play an important role in organ or tissue development, cell differentiation and apoptosis, cell cycle control, cellular transport, metabolic processes, chromosome dynamics, and so on [16,17,18,19].

Since ncRNAs are so important in various biological processes, more and more evidences have been found to reveal the associations between ncRNAs and human diseases [7,8,20,21]. Many ncRNAs have been reported as both oncogenes and tumor suppressors in the tumorigenesis of diverse cancers [22,23,24,25]. Therefore, ncRNAs were regarded as important biomarkers in the diagnosis, treatment and prognosis of complex diseases, and furthermore as potential drug targets [26,27,28,29]. The work on predicting ncRNA–disease associations is significant for human disease diagnostics and prognostics, and will improve the development of drug discovery. Several effective computational models have been developed for the prediction of potential ncRNA–disease associations and small molecule–ncRNA interactions [30,31,32,33,34].

This Special Issue is composed of twelve papers, including ten original researches [35,36,37,38,39,40,41,42,43,44] and two review articles [45,46]. The topics of these manuscripts involve diverse ncRNAs, such as miRNA, lncRNA and circRNA, related to specific disease or different diseases. Besides computational studies, some experimental works were included to enrich the content of this issue.

Pan et al. [35] applied machine learning algorithms to analyze the expression patterns of snoRNA in eight tumors. They used the Monte Carlo Feature Selection (MCFS) to analyze the expression level of snoRNAs and select the informative features. Then, based on the optimized features by incremental feature selection (IFS), the support vector machines (SVMs) were employed to classify different cancer types.

Peng et al. [36] developed a novel computational model named RPITER to predict potential ncRNA–protein interactions in a hierarchical deep learning framework. In this model, they first processed the sequence data via improving the conjoint triad feature (CTF) coding method. Then, the convolution neural network (CNN) and stacked auto-encoder (SAE) were adopted to construct the predicting framework. The validation results showed the good performance of RPITER.

In the work of Zhao et al. [37], an effective model was presented for the prediction of miRNA-disease association. They first built a weighted interactive network using disease similarity, miRNA similarity and known miRNA–disease associations. Then, the weighted interactive network-based model for miRNA–disease association inference (WINMDA) was developed by considering both the *T* nearest neighbors and the shortest path algorithm.

Wang et al. [38] incorporated several datasets, including circRNA, mRNA, miRNA and pathway data to design a non-negative matrix factorization-based model for identifying breast cancer-related circRNA modules. As a result, they discovered 13 circRNA modules associated with breast cancer.

Chen et al. [39] studied the tissue specificity of lncRNA and investigated the difference from mRNA. Several feature selection approaches were implemented to choose important genes for both lncRNA and mRNA. The random forest (RF) and the repeated incremental pruning to produce error reduction (RIPPER) algorithm were subsequently employed as two classifiers. The analysis results indicated that lncRNAs were distinguished from mRNAs on expression pattern.

Pewarchuk et al. [40] screened new miRNAs associated with gastric adenocarcinoma (GA). Based on small RNA sequencing data, they used the miRMaster platform to analyze and identify 170 novel miRNAs that were expressed specifically in GA. Moreover, the combined expression of two novel and one annotated miRNAs was found to be related to patient outcome.

In the manuscript of Pan et al. [41], a novel lncRNA named *interferon-stimulated lncRNA* (*ISR*) was reported to be implicated in influenza A virus (IAV) infection. They revealed that silent or ectopic expression of *ISR* could increase or reduce the replication of IAV in infected cells.

In order to answer the question whether the miRNAs regulate genes in a random pattern or the genes are regulated referring to their functions, Mustafa et al. [42] investigated this problem in cardiometabolic disorders by performing an enrichment analysis. The result of their research provided support to the non-random regulation of miRNAs to genes.

Xu et al. [43] analyzed gene expression and co-expression to construct a gene network module for thyroid cancer, based on which a novel drug selection method was proposed for reversing the perturbations of thyroid cancer.

Chi et al. [44] focused on the function of lncRNA in bladder cancer (BC). They utilized the microarray assay to compare the expression profiles of lncRNA and mRNA in BC. As a result, the lncRNA *RP11-79H23.3* was discovered, which could sponge *hsa-miR-107* to regulate the expression of phosphatase and tensin homolog (PTEN) in BC.

In the review paper of Zhang et al. [45], some important results about the lncRNA–protein interactions, including relevant databases, experimental approaches and computational models, especially those based on networks, were combed clearly. In addition, Zhao et al. [46] summarized the kinetic modeling methods that investigated the association of miRNA-mediated signaling networks and diseases.

In conclusion, papers contained in this Special Issue will benefit people to deeply understand the relations between ncRNAs and human diseases. It will also improve the development of computational models on the hot topics of related fields. We want to thank the contributions from all authors, the hard work of all editorial staff members as well as the dedication of all the anonymous reviewers. We hope this issue will be helpful and interesting to readers.
